# Quantification of Movement Error from Spiral Drawing Test

**DOI:** 10.3390/s23063043

**Published:** 2023-03-11

**Authors:** Hyunjin Yoon, Minkyu Ahn

**Affiliations:** School of Computer Science and Electrical Engineering, Handong Global University, Pohang 37554, Republic of Korea

**Keywords:** movement error, movement disorder, Parkinson’s disease, essential tremor, spiral drawing

## Abstract

Parkinson’s disease is a neurodegenerative disease that often comes with symptoms such as muscle stiffness, slowness of movement, and tremors at rest. Since this disease negatively influences the quality of life in patients, an early and accurate diagnosis is important for slowing the progression of the disease and providing effective treatment to patients. One of the quick and easy methods for diagnosing is the spiral drawing test and the differences between the target spiral picture and the drawing by patients can be used as an indicator of movement error. Simply, the average distance between paired samples of the target spiral and the drawing can be easily calculated and used as the level of movement error. However, finding the correct pair of samples between the target spiral and the drawing is relatively difficult, and the accurate algorithm to quantify the movement error has not been thoroughly studied. In this study, we propose algorithms applicable to the spiral drawing test, that ultimately can be used to measure the level of movement error in Parkinson’s disease patients. They are equivalent inter-point distance (ED), shortest distance (SD), varying inter-point distance (VD), and equivalent angle (EA). To evaluate the performance and sensitivity of the methods, we collected data from simulation and experiments with healthy subjects and evaluated the four methods. As a result, in normal (good drawing) and severe symptom (poor drawing) conditions, the calculated errors were 3.67/5.48 from ED, 0.11/1.21 from SD, 0.38/1.46 from VD and 0.01/0.02 from EA, meaning that ED, SD, and VD measure movement error with high noise while EA is sensitive to even small symptom levels. Similarly, in the experiment data, only EA shows the linear increase of error distance to changing symptom levels from 1 to 3. In summary, we found that EA is the most effective algorithm in finding the correct pair of samples between the spiral and the drawing, and consequently yields low errors and high sensitivity to symptom levels.

## 1. Introduction

Parkinson’s disease [1,2] is a neurodegenerative disease that causes muscle stiffness, slow motion, and tremors at rest. Since it occurs mainly in the elderly, sometimes patients may miss the right time for the correct diagnosis of this disease [3]. Considering that this disease worsens and influences the quality of a patient’s life, early and accurate diagnosis is very important for providing the right treatment to the patient at the right time.

The methods for diagnosing this disease are mostly based on the patient’s medical history, symptoms of abnormal movement, and the degree of loss of living ability. The process of diagnosis often goes with questionnaires such as the Unified Parkinson’s Disease Rating Scale (UPDRS) or the Clinical Rating Scale for Tremor (CRST). However, these methods may be slow and could be subjective [4,5]. Thus, a more intuitive and easier procedure that can give an accurate and immediate result is required for continuous monitoring of disease. A drawing test as included in CRST can be the candidate since it can be performed easily by patients anywhere and anytime. For example, a patient can draw a spiral pattern by using a pen and paper. Then, the drawn image can be examined by a clinician for diagnosis. However, this process is also somewhat manually performed. Thus, the diagnosis can be subjective, and the final decision may vary depending on the examiner. Therefore, it is necessary to develop a method for evaluating the symptom level of the patients, objectively and accurately. Many researchers are currently making attempts at diagnosing movement symptoms in Parkinson’s disease [6,7]. In recent studies, we also developed a tablet PC-based drawing application that can be easily used by clinicians [8,9]. We expressed the spiral drawing test with exact (x,y) coordinates. Since the spiral drawing test was implemented digitally, all information is saved, and the ‘Error Distance’ between the target and drawn patterns can be calculated with a certain algorithm. 

The purpose of this study is to present quantification algorithms from the spiral drawing test that can be used for an accurate diagnosis of movement error and to evaluate the performance of the algorithms. Therefore, a study was conducted to increase the accuracy of the ‘Error Distance’ in poor to good drawings [10]. The ‘Error Distance’ determines how far the person’s drawing deviates from the baseline of the presented straight or spiral figure. Therefore, to calculate the error distance, it is important to find the correct coordinates of the baseline based on the drawing data.

There are various possible methods of calculating Error Distance. The most common method is to calculate the distance by sequentially comparing the baseline and the drawing. For accurate ‘Error Distance’ calculation, the same number of baseline data as (x,y) coordinates are required. However, although this method is effective in drawing a straight line, an error occurs in the process of comparing patient data and baseline coordinates of spiral drawing. When a patient with movement disorder performs the spiral drawing test, the spacing between the (x,y) coordinates is influenced by the speed of drawing. That is, when the patient draws a line faster, the interval between the coordinates becomes wide, and when the patient draws a line slower, the interval becomes narrower. Therefore, even if the length of the baseline and the patient’s (x,y) coordinates are the same, they exist at different angles because the distance between the two coordinates is not constant. As a result, it is difficult to provide an inspection result by measuring the exact ‘Error Distance’ because it cannot be paired with the exact position on the coordinates.

In this study, we propose algorithms that can accurately measure the ‘Error Distance’ of movement error in the spiral drawing test by finding the optimal pairs of points between the base and the drawing of the spiral pattern. In the following sections, we introduce the methods for finding coordinate pairs and propose an algorithm. Afterward, the evaluation strategy with simulation and healthy subjects’ experiments is explained. Finally, we report the performance of the three methods and our proposed algorithm for estimating error distance.

## 2. Materials and Methods

### 2.1. Methods for Movement Error Quantification

#### 2.1.1. Problem Definition

Measuring error distance can be modeled as follows. Let $B(i)\in {\mathbb{R}}^{2}$ and $D(j)\in {\mathbb{R}}^{2}$ two coordinates ($B_{x}(i)$, $B_{y}(i)$) of ith sample in the base spiral pattern and ($D_{x}(j)$, $D_{y}(j)$) of jth sample in the drawing by a patient. Indices are denoted as i∈{1,…,b} and j∈{1,…,d}, b and d are the last indexes or the number of samples in each data. Normally, d≪b since the spiral pattern is generated with any resolution while the number of points of the drawing depends on the sampling rate in a digital device (e.g., tablet PC), the error distance e is calculated by averaging the distance between pairs of B(k) and D(k) over samples (k∈{1,…p}), as $e=<\left\|B\left(k\right)-D(k)\right\|>$. Here, <·> and $\left\|\cdot \right\|$ denote the average over samples and the L2 norm of the given vector. By substituting $i_{k}$ and $j_{k}$ with k for simplicity, $B\left(k\right)=B(i_{k})$ and $D\left(k\right)=D(j_{k})$, and the number of points p in the pairs satisfy p≤b,d. Finally, the question is how to find the correct pair $B(i_{k})$ and $D(j_{k})$.

#### 2.1.2. Equivalent Inter-Point Distance (ED)

**Figure 1:**
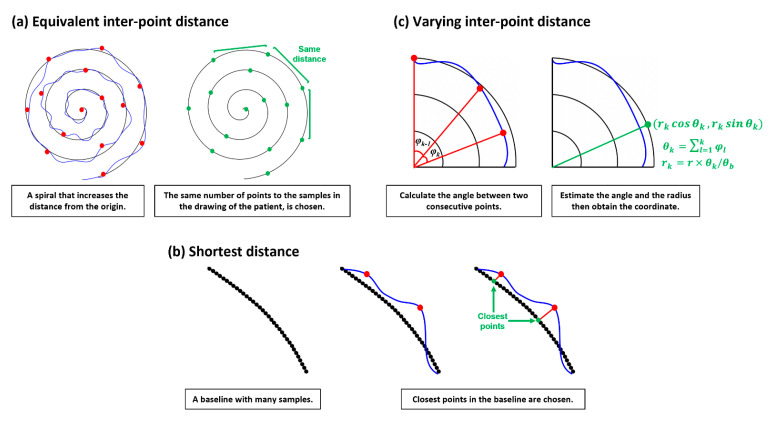
Three approaches for error distance calculation.

The easy and intuitive way to find the pairs of points between the base and drawing patterns is selecting by every nth sample. This assumes that the drawing likely starts from the first position and ends at the last position of the given base spiral pattern, thus, applying $i_{k}=1+(k-1)n$ and $j_{k}=1+(k-1)m$, $B\left(k\right)=B(i_{k})$ and $D\left(k\right)=D(j_{k})$. The constant intervals n and m between samples can be estimated from n=(b−1)/p and m=(d−1)/p with a given p≤d. This approach is illustrated in Figure 1a. However, if the drawing by a patient largely fluctuates or does not end at the last position of the base spiral, then the coordinate pairs may be incorrect. Consequently, the estimated ‘error distance’ becomes unreliable.

#### 2.1.3. Shortest Distance (SD)

Normally, the spacing between samples in the drawing is not constant, while it can be constant in the base pattern since this is generated. This mismatch may introduce large errors in finding pairs. Whereas pairing the closest point in the base with the target point in the drawing may be more reasonable and the whole sample (k=j and p=m) in the drawing can be used in estimating ‘error distance’, this assumes that a patient probably tries to follow the given base pattern, yielding two points in a likely close pair. Applying this notion, the paired point in the base with the kth sample in the drawing can be found by $i_{k}=\underset{i}{\min}\left\|B(i)-D(k)\right\|,i_{k}<i_{k+1}$. This approach is described in Figure 1b. 

#### 2.1.4. Varying Inter-Point Distance (VD)

Since a patient will follow the given base pattern that rotates with the increasing length of the radius, the change of angle from a point to a point may be also useful information. Thus, the paired point $B(i_{k})$ in the base can be found by reflecting the change of the angle between the two vectors at D(k−1) and D(k). This between-angle $\varphi_{k}$ can be estimated from the two points $D\left(k-1\right)$ and $D\left(k\right)$ in the drawing by the raw of cosign as below.

(1) $$\varphi_{k}=\cos^{-1}\left[\frac{\left({\left\|D\left(k-1\right)-D\left(k\right)\right\|}^{2})-({\left\|D\left(k-1\right)\right\|}^{2}+{\left\|D\left(k\right)\right\|}^{2}\right)}{2\left\|D\left(k-1\right)\right\|\left\|D\left(k\right)\right\|}\right]$$

Subsequently, the cumulative angle $\theta_{k}$ from the original plane at θ=0 and the estimated radius $r_{k}$ at the point can be obtained by $\theta_{k}=\sum_{l=1}^{k}\varphi_{l}$ and $r_{k}=r\times \theta_{k}/\theta_{b}$ where normally $\theta_{b}\approx 6\pi$ and r is the largest radius in the base spiral. Finally, we can calculate the paired point $B\left(i_{k}\right)=(r_{k}\cos\theta_{k},r_{k}\sin\theta_{k})$. Unlike ED, this method could reflect the way a patient draws. However, errors may occur when the patient moves backward, then forward in direction due to tremors. In this case the $\varphi_{k}$ can be poorly estimated.

#### 2.1.5. Equivalent Angle (EA)

**Figure 2:**
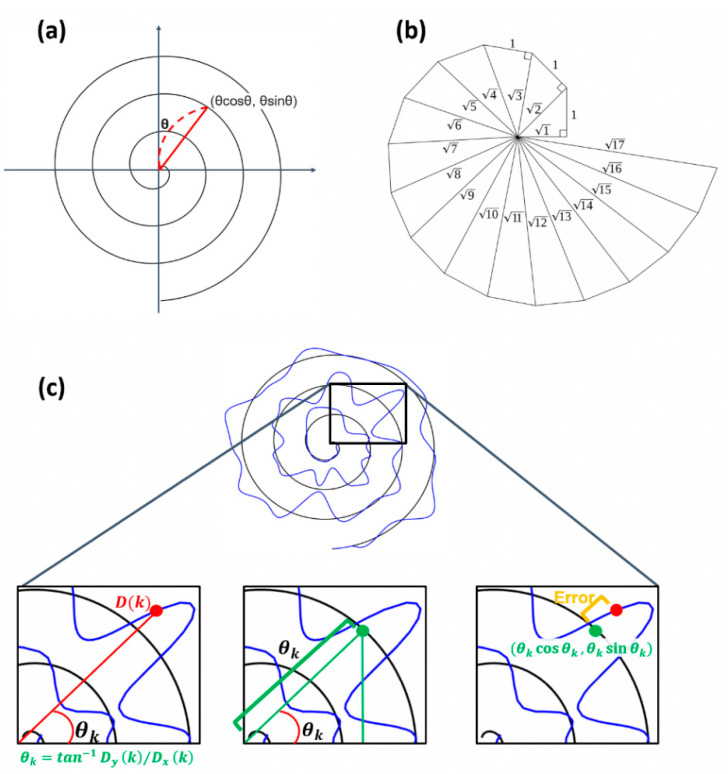
Illustration of the procedure calculating error distance using EA. (**a**) Example of Archimedean spiral. (**b**) Angle within the spiral. (**c**) Description of how the proposed algorithm works.

We propose a method utilizing the characteristics of the Archimedean spiral to obtain pairs for estimating more accurate movement errors. As shown in Figure 2a,b, the Archimedean spiral is a line moving away from the fixed origin at a constant speed and an angular velocity [11]. The spiral can be described as r=aθ in polar coordinates, and the distance between loops can be controlled by the real number a. By setting a = 1, then the coordinate of each point in the Archimedean spiral is (θcos⁡θ,θsin⁡θ). By using this characteristic, the paired point in the base can be found. 

With the assumption that the spiral rotates 3 times, meaning the angle θ between the *x*-axis and the coordinate of a point is within [0,6π], the θ at $D\left(k\right)$ can be calculated as $\theta_{k}=\tan^{-1}D_{y}(k)/D_{x}(k)$. The point having the same angle θ in the base spiral is chosen as the paired point with $D\left(k\right)$ as follows: ${B(i}_{k}=i)$ satisfying $\tan^{-1}\frac{B_{y}\left(i\right)}{B_{x}\left(i\right)}=\theta_{k},i_{k}<i_{k+1}$. 

### 2.2. Simulation Study

**Figure 3:**
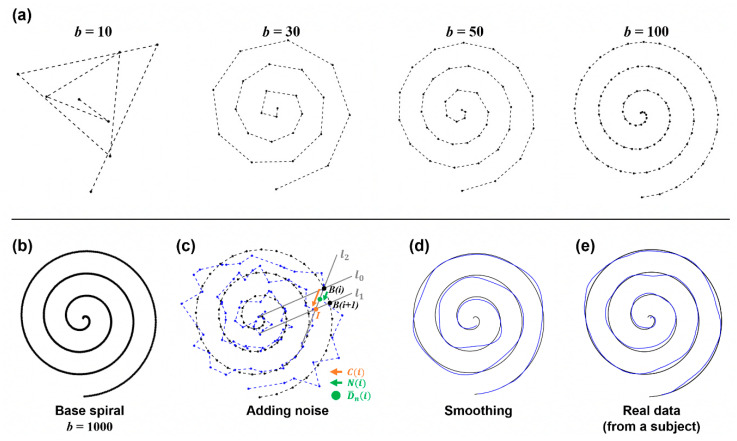
Principles and process of simulation data. (**a**) The base spiral generation at a different number of points. (**b**) Base spiral at b=1000 that is used in this study. (**c**) Illustration of adding noise. At B(i), N(i) with a noise level L was chosen and added to B(i). (**d**) The result after smoothing. (**e**) For comparison, the real data acquired from a representative subject are shown.

For validation of the methods, we conducted a simulation study. Synthesized patient data were modeled and 100 instances of data with 10 different error distances were generated. To these data, we applied the four methods and quantified error distance based on the detected pairs between points from the base and the drawing of the spiral pattern. This simulation was performed in MATLAB, and we created synthesized patient data based on the following steps as described in Figure 3.

#### 2.2.1. Base Spiral Generation

A spiral can be drawn from the central point by gradually increasing the angle θ and the radius of a corresponding circle. By following this process, we generated the sample points of the base spiral pattern. Therefore, the coordinate B(i) of each sample in the base spiral was calculated using a trigonometric function of the hypotenuse r and the angle θ between two points on the helix from the center point (0, 0). Thus, $B\left(i\right):\left(B_{x}\left(i\right),B_{y}\left(i\right)\right)=\left(r_{i}\cos\theta_{i},r_{i}\sin\theta_{i}\right),i\in \left\{1,\ldots ,b\right\}.$ At each point i, we increase θ and r. r is calculated by dividing the radius of the final spiral and applied by dividing it by the number of samples. θ is calculated by setting it to 6π from 0.

As the number (b) of samples increases, the base spiral with higher resolution can be obtained, as seen in Figure 3a,b. In this simulation, b was set to 1000 to insure sufficient resolution.

#### 2.2.2. Adding Movement Error

Next, the noise was added to the base spiral to generate the synthesized drawing data $\tilde{D}(i)$. The random noise $N_{L}\left(i\right):\left(N_{L,x}\left(i\right),N_{L,y}\left(i\right)\right)$ was simply added to $B\left(i\right)$. For this process, we predefined 10 noise levels as $N_{L,x}\left(i\right),N_{L,y}\left(i\right)\in L\times [-100,100]$ where an integer L∈[1,10].

(2) $${\overset{-}{D}}_{n}(i):({\overset{-}{D}}_{n,x}(i),{\overset{-}{D}}_{n,y}(i))=\left(B_{x}\left(i\right)+N_{n,x}\left(i\right),B_{y}\left(i\right)+N_{n,y}\left(i\right)\right)$$

However, to prevent the occurrence of too large and unrealistic noise, the random noise was selected to satisfy the condition $\left\|N_{L}\left(i\right)\right\|<\left\|C(i)\right\|$ where C(i) is the vector between B(i) and the intersection point (I) of two lines $l_{1}$ and $l_{2}$ in Figure 3c. 

#### 2.2.3. Smoothing

The simple addition of noise to the base spiral may introduce disconnectedness to data, yielding a generated sample that looks artificial. To make the data smoother and more realistic, sample-wise smoothing was applied by averaging samples and a low-pass frequency filter. The cutoff frequency for low pass filtering was set to 3 Hz, since normal tremor (one of the symptoms of movement disorder) is presented within 3–15 Hz, and the motion component is likely under 3 Hz [12]. This step removed the discontinuity from ${\overset{-}{D}}_{n}(i)$ and generated smoothed data ${\tilde{D}}_{n}(i)$ as seen in Figure 3d. 

#### 2.2.4. Expressing the Speed

When a real patient performs a drawing test, the measurement speed continuously changes due to bradykinesia or rigid movement. This feature was also reflected in the sample data. First, the whole series of sample points were divided into several sections, then different speeds were expressed for each section. This process was implemented by choosing several sample indices within each section and removing them from the total samples. Finally, we obtained the sample data ${\tilde{D}}_{n}(i),i\in \left\{1,\ldots ,d\right\}\;where\;d<b$.

### 2.3. Experiment

This experiment aims to obtain a dataset at different movement error levels simulated by human subjects, thereby comparing and analyzing the performance of each algorithm described above for further evaluation. For this purpose, four healthy subjects (age: 20–25 years old, 4 males) participated in the spiral drawing experiment. None of the subjects had participated in a spiral drawing experiment before this study. All subjects were informed of the purpose of the study, the subject’s rights, and possible rewards. A written consent form was acquired from all subjects, then the experiment was performed. For better simulation by subjects, we provided a sufficient explanation of motor symptoms in Parkinson’s disease, possible movement error, and the spiral drawing test. This experiment was approved by The Public Institutional Bioethics Committee designated by the Ministry of Health and Welfare approved study (P01-202012-13-002).

**Figure 4:**
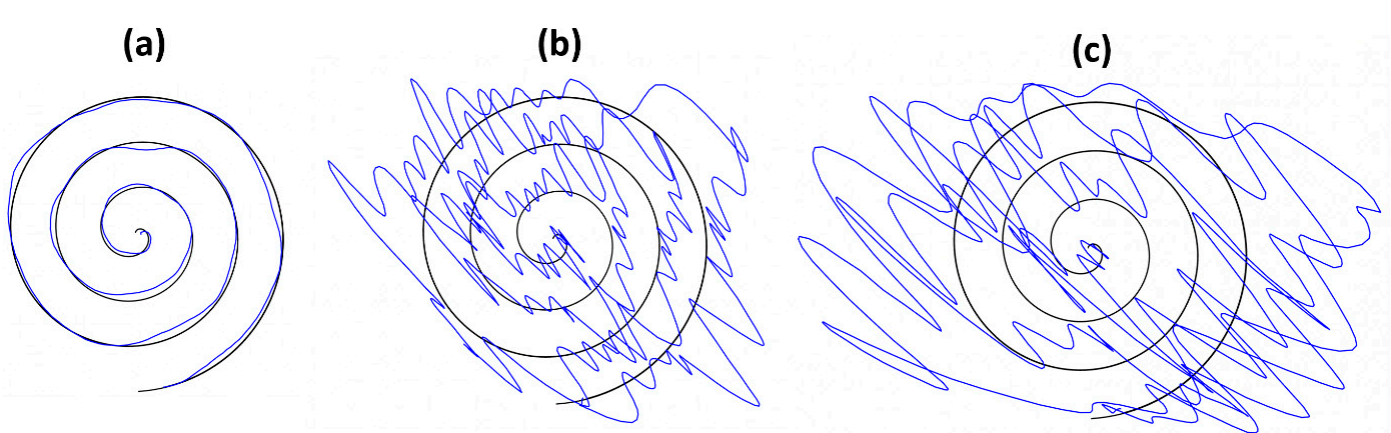
Representative spiral drawings. (**a**) normal condition, (**b**) mild symptoms, and (**c**) severe symptoms.

The experiment was designed to acquire drawing samples of three different conditions (normal, mild, and severe movement symptoms). Thus, each subject was asked to draw a spiral pattern according to each condition and produced 15 sample drawings per condition on a tablet PC. The representative sample drawings of three conditions (normal, mild, and severe) are shown in Figure 4. As a whole, we collected 45 sample drawings per subject. Each experiment took a total of 90 min and drawings were recorded in the form of a CSV file.

### 2.4. Data Analysis and Statistical Tests

We obtained 1000 instances of synthesized data (10 conditions and 100 samples per each condition) and 180 pieces of experiment data from four subjects; each subject conducted 15 times per each condition and there were 3 conditions. With these data, we obtained paired samples between the base spiral and the drawing and calculated the error distance by applying four algorithms (ED, SD, VD, and EA). We then compared the performance and reliability of the methods. Throughout this study, sample groups were compared, and the significance was checked. We used the Wilcoxon Rank Sum Test as the statistical test in this study because we do not know if the samples follow the normal distribution.

## 3. Results

In this section, we present the representative drawing and its obtained pairs of samples from four algorithms, and the results from comparative analysis based on the above simulation data and experiment data. 

### 3.1. Drawing Results

**Figure 5:**
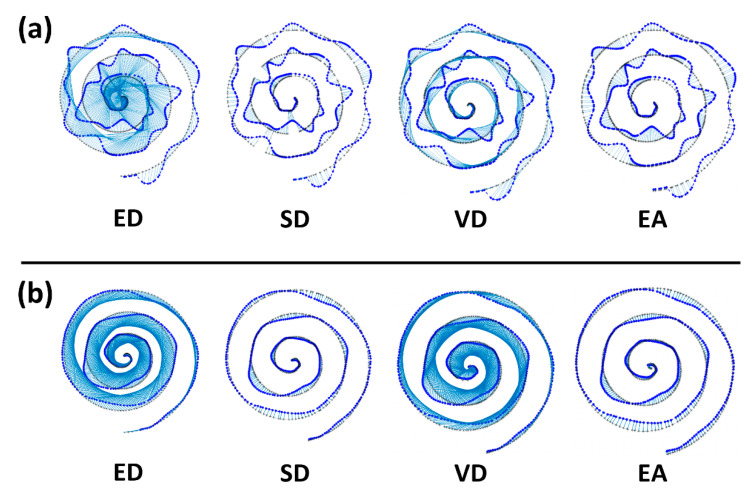
Representative drawing results of simulation (**a**) and experiment (**b**). In each figure, the base spiral pattern (black) and the drawing (blue) are presented, and estimated pairs between the spiral and the drawing are marked in light blue lines. Some meaningful mismatches are marked with blue rectangles.

Figure 5 shows the results of one representative drawing from the simulation and experiment. The estimated pairs between the base spiral and the drawing are connected by light blue lines for better visualization. This helps to identify the mismatches of pairs and simply the total length of blue lines can be seen as the estimates of error in each figure. As shown, more mismatches of pairs are observed in ED, SD, and VD compared to the results of EA. We obtained that the total length is 376.53 cm for ED, 30.65 cm for SD, 280.96 cm for VD and 20.28 cm for EA in Figure 5a and 304.92 cm for ED, 18.83 cm for SD, 252.21 cm for VD and 13.74 cm for EA in Figure 5b. EA showed the shortest length of blue lines, meaning that the error is smaller than with other methods.

### 3.2. Accuracies of Methods

**Figure 6:**
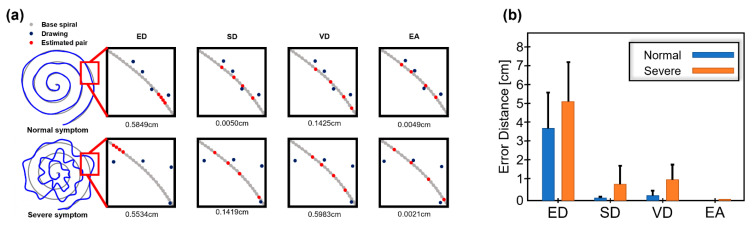
Differentiation of symptom levels in experimental data. (**a**) Examples of normal and poor drawings and estimated pairs from four methods are presented. (**b**) The error errors are shown in mean and standard deviation.

Next, we evaluated each method to check if the method well differentiates symptom levels from drawings. In Figure 6a, the two example drawings from the normal and severe symptoms (or good and poor drawings) are shown, and the estimated pairs are shown in each subfigure. It is easily observable that samples of the drawing are far from the samples of base spiral in severe symptoms (poor drawing), and consequently, it is likely to find incorrect pairs except for the method EA.

In methods ED and VD, the pair values were significantly different in both cases, and SD seems to find partially correct pairs in the normal symptom, but not in severe symptoms. In comparison, EA finds pairs in both normal and severe symptoms with a small difference. The pair error was calculated per each method and presented in Figure 6b.

As a result, in normal (a good drawing) and severe symptom (a poor drawing), the calculated errors are 3.67 ± 2.52/5.48 ± 2.33 from ED, 0.11 ± 0.04/1.21 ± 1.53 from SD, 0.38 ± 0.30/1.46 ± 1.19 from VD and 0.01 ± 0.01/0.02 ± 0.02 from EA. Statistical tests revealed that all methods differentiate the two levels of drawing quality (or symptoms), showing *p* < 0.05.

**Figure 7:**
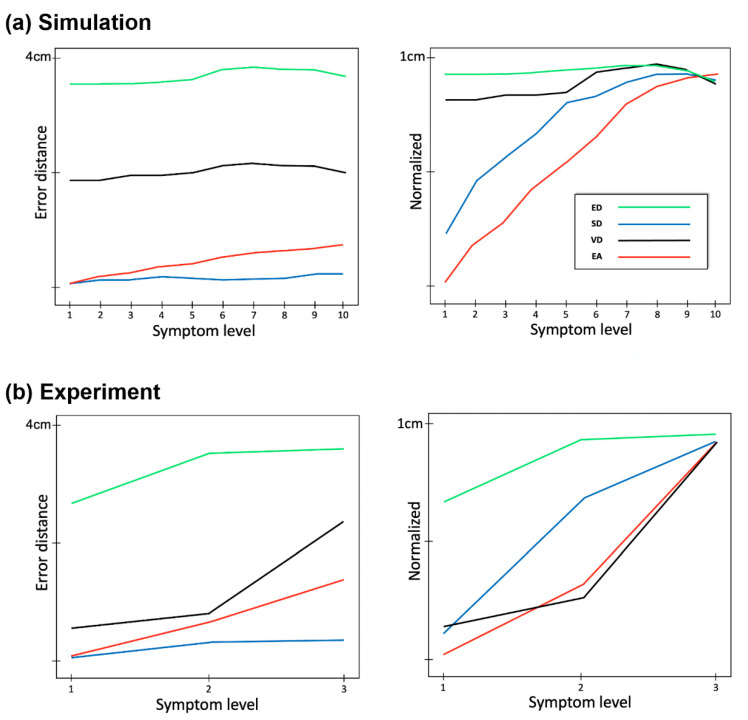
Comparison of error distances for each error stage between experimental data and simulation data. Error distance is shown as a function of symptom level from normal to severe in simulation data with 10 levels (**a**) and experiment data with 3 levels (**b**). The figures on the right are the normalized results. For all cases, the best and the worst levels show significant differences (*p* < 0.05, from Wilcoxon Rank Sum Test).

For additional verification of method performance, a comparative analysis was performed on simulation and experimental data. Error distance for four methods based on generated data and measurement data is shown in Figure 7. As expected, the more severe the symptom is, the higher the error distance is. However as shown in Figure 7a, it seems that ED, SD, and VD are not sensitive to the small differences in symptom levels, while EA shows a relatively highly correlated and sensitive result to symptom levels. Similarly, in experiment data, only EA shows the linear increase of error distance to changing symptom levels from 1 to 3.

## 4. Discussion

The drawing error in the spiral drawing test can be an effective measure for diagnosing movement disorders [13]. In measuring drawing error, the error distance between samples of the target base spiral and a drawing by the patient is one good measure. Consequently, finding the correct pair of samples between the target spiral and the drawing pattern is important for estimating accurate error distance. In this study, we designed four methods and evaluated their performance with simulation and experiment data. As a result, we observed that EA shows a relatively smaller error scale compared to other methods and was sensitive to slight changes in drawing quality (by symptom severity). From these results, we think that EA is suitable for finding the correct pairs between the base spiral and the drawing and consequently yields accurate error distance.

Generally, spiral drawing tests are conducted with a pen and paper. This requires manual checking by a clinical expert. With the help of various digital devices (e.g., tablet PC), the patient’s drawing data can be easily collected in digital format so that objective evaluation can be made by algorithms as proposed by this study. In this sense, we think that our study and results are meaningful. From the application perspective, our algorithm with spiral drawing test can be applied to understand the possible differences in movement error across different movement disorders, for example, Parkinson’s disease. In addition, the accurate error distance may be combined with brain MRI [14] to improve the accuracy of the diagnosis of movement disorder.

We believe that error distance with EA is effective for evaluating movement error. However, there are still limitations to this study. First, normal movement error may be caused by various factors like slowness or rigidity of movement and tremors. We did not consider each of these factors in designing the algorithm. With a more sophisticated algorithm based on an understanding of such error factors, the evaluation accuracy may be improved. Second, we conducted simulation and experiment studies for reliability testing, but the algorithms should be tested with true patient data. We will pursue these as our future research topics.

## 5. Conclusions

We proposed algorithms to accurately quantify the error distance of a spiral drawing test that is important for the diagnosis of movement disorder. The algorithms were evaluated with simulation and experiment data, and we have shown the performances of the algorithms. As a result, EA, which uses an ‘Equivalent Angle’ in finding the correct pair of samples between two drawings, is proven to be the best among the proposed methods. We believe that this algorithm could be effectively used for objective diagnosis of movement disorder.

## Data Availability

The data presented in this study are available on request from the corresponding author.
